# Multiplex immunofluorescence assessment of macrophages and IL-23R in inflammatory and malignant diseases of the oral mucosa: a pilot study

**DOI:** 10.3389/fimmu.2025.1569490

**Published:** 2025-04-14

**Authors:** Leah Trumet, Bettina Grötsch, Abbas Agaimy, Kerstin Galler, Carol Geppert, Linus Winter, Jutta Ries, Marco Kesting, Manuel Weber

**Affiliations:** ^1^ Department of Operative Dentistry and Periodontology, Friedrich-Alexander-Universität Erlangen-Nürnberg (FAU), Erlangen, Germany; ^2^ Deutsches Zentrum Immuntherapie (DZI) and Comprehensive Cancer Center Erlangen-EMN (CCC ER-EMN), Friedrich-Alexander-Universität Erlangen-Nürnberg (FAU), Erlangen, Germany; ^3^ Department of Internal Medicine 3-Rheumatology and Immunology, Friedrich-Alexander-Universität Erlangen-Nürnberg (FAU), Erlangen, Germany; ^4^ Institute of Pathology, Friedrich-Alexander-Universität Erlangen-Nürnberg (FAU), Erlangen, Germany; ^5^ Department of Oral- and Cranio-Maxillofacial Surgery, Friedrich-Alexander-Universität Erlangen-Nürnberg (FAU), Erlangen, Germany

**Keywords:** OSCC, periodontitis, oral cancer, oral medicine, Lichen Planus, leukoplakia, N+, lymph node metastases

## Abstract

**Background:**

Immune cells play a major role in the development and progression of inflammatory and malignant diseases of the oral mucosa. There is growing evidence that immune cells contribute to oral cancer progression and metastases. Inflammatory carcinogenesis is believed to be relevant for oral Lichen Planus as well as for oral Leukoplakia. In addition, there is growing evidence that periodontitis might also be linked to oral cancer development. Yet there is no analysis available comparing the immune cell composition in these different inflammatory and malignant neoplastic diseases. A better understanding of similarities and differences of the diseases could eventually also pave the way for the use of immunotherapy in non-malignant diseases.

**Methods:**

In the current pilot study, a tissue microarray (TMA) was created of a total of 29 patients with periodontitis (PD, n=4), oral Leukoplakia (OL, n=4), oral Lichen Planus (OLP, n=4), oral squamous cell cancer without lymphatic metastases (OSCC N0, n=5), or with lymphatic metastases (OSCC N+, n=4), OSCC biopsies prior to and resection specimens after anti-PD1 immunotherapy (IT) (each n=3) as well as healthy control gingiva (n=5). In each patient two tissue samples were analyzed. The TMA was stained with a 4X multiplex immunofluorescent staining for IL-23R, CD68, CD11c, and CD163. Samples were digitalized and an AI-based cell counting was performed. Statistical analysis was performed using the Mann-Whitney U test.

**Results:**

IL-23R expression, macrophage infiltration as well as M2 polarization in OL and OLP were significantly higher compared to controls. OLP showed a significantly higher M2 infiltration and polarization than OL. PD showed a trend for increased macrophage infiltration compared to controls without significance. N+ OSCC showed a significantly increased macrophage infiltration compared to N0 cases. In response to anti-PD1 IT, CD11c and CD163 infiltration was significantly increased. Most IL-23R positive cells co-expressed macrophage markers.

**Conclusion:**

A TMA in combination with 4-plex immunofluorescence is suitable for immune cell characterization in different oral diseases. Macrophage infiltration and polarization in precursor lesions seems to be associated with OSCC development as well as metastatic spread. IL-23 pathway inhibition might be a potential target for oral Lichen and Leukoplakia.

## Introduction

Oral squamous cell carcinomas (OSCC) account for 95% of oral cancers, with most cases believed to arise from benign and inflammatory precursor lesions such as Oral Leukoplakia or Oral Lichen Planus (1). Of note, also chronic inflammation in Periodontitis elevates inflammatory cytokines, which strongly stimulate the proliferation and fusion of myeloid precursors, as well as their resistance to cell death. This suggests that periodontal disease might also play a significant role in promoting tumor cell transformation in oral premalignant lesions (2).

The critical role of the tumor microenvironment (TME) in the initiation, development, and progression of OSCC is well-established. Within the TME, immune cells like tumor-associated macrophages (TAMs), cancer-associated fibroblasts, T-lymphocytes, tumor-associated neutrophils, myeloid-derived suppressor cells, and dendritic cells (DCs) form a complex cellular network that influences OSCC proliferation, invasion, migration, and angiogenesis (3).

Macrophages are shown to play an important role in a wide range of inflammatory and malignant diseases. Due to their role in inflammation and in tumor progression, they could also be critical cells involved in immune mediated processes of malignant transformation. Macrophages are highly plastic cells that can execute vastly different functions dependent on the environment and their activation set (4). In this regard, the polarization of the macrophages is believed to be one of the most important factors. They appear either as M1 polarized cells that act pro-inflammatory in response to pathogenic stimuli or as M2 polarized macrophages that promote tissue regeneration, wound healing and angiogenesis. In the context of malignant diseases, M1 polarized macrophages are considered as anti-tumoral, whereas tumor-promoting capabilities are attributed to M2 macrophages (4). Surface markers to identify these subtypes are CD68 for resting (M0) macrophages and M1 polarized cells, CD163 for M2 polarized macrophages and CD11c, a marker for DCs, which is - to some extent - suitable to detect M1 polarized cells (4–6).

Notably, the balance between pro-inflammatory, anti-tumorigenic (M1-like) macrophages and anti-inflammatory, pro-tumorigenic (M2-like) macrophages is crucial in supporting tumor progression also in OSCC (3). There is evidence of an increased shift towards M2 polarized macrophages in OSCC with lymph node metastases (7). In addition, an increased infiltration of macrophages as well as a switch towards M2 polarization has been shown to be associated with malignant transformation of Oral Leukoplakia, thus showing their potential role also in the initiation of malignancies (6).

IL-23 is a pro-inflammatory cytokine that is primarily produced by DCs and activated macrophages (8). Under physiologic conditions, IL-23 signaling seems to contribute to a proper antimicrobial immune response. Patients with autosomal recessive IL-23R deficiency showed susceptibility to mycobacterial disease and mucocutaneous candidiasis (9). IL-23 signaling plays an important role in the pathogenesis of Crohn’s disease, spondylarthritis and psoriasis, among others (9). In these diseases, IL-23 inhibition is successfully used as a therapeutic approach (9). The receptor IL-23R is described to be expressed by several immune system cells, including T-cells, natural killer cells (NK cells), macrophages and DCs (8). On T-cells, IL-23R signaling leads to the induction of a Th17 phenotype which is believed to contribute to the pathogenesis of periodontitis (10) In contrast, the role of IL-23R activation on other immune cells is poorly understood (8). In addition, IL-23 has been shown to promote tumor growth in preclinical cancer models and is linked to poor clinical outcomes. Interestingly, IL-23 produced by tumor-associated macrophages appears to stabilize T_reg_ cell identity, thereby enhancing immunosuppression and promoting tumor growth (11). However, the role of the IL-23/IL-23R axis in tumor progression remains poorly understood.

Immunotherapy (IT) with anti-PD1 antibodies has become a routine therapy in advanced OSCC cases without surgical or radiotherapeutic treatment options. Recent phase-2 studies indicate promising results if anti-PD1 IT is neoadjuvantly applied prior to primary surgical treatment (12). Besides tumor cells itself, macrophages are a main source of PD-L1 expression in tumor tissue (13). It is yet unclear how macrophage infiltration and polarization changes in response to anti-PD1 IT. Interestingly, there are first clinical studies conducted applying IT even in OSCC precursor lesions like Oral Leukoplakia (14), even if the basic biologic principles of immune mediated malignant transformation are not yet understood.

The current pilot study aimed to characterize macrophage infiltration and IL-23R expression in oral mucosa both in inflammatory diseases as well as different malignant pathologies, healthy oral mucosa served as a negative control. In addition, OSCC cases with and without lymph node metastases as well as cases receiving neoadjuvant IT were compared. Therefore, samples were collected from patients with periodontitis, oral leukoplakia and oral Lichen Planus and from patients treated for different stages of OSCC before and after anti-PD1 immunotherapy.

## Materials and methods

### Study design

The following groups were established for this pilot study and mucosa was collected from patients with:

1. Healthy Gingiva.
2. Severe Periodontitis (PD).
3. Oral Leukoplakia (OL).
4. Oral Lichen Planus (OLP).
5. OSCC Resection specimens (N0 cases).
6. OSCC Resection specimens (N+ cases).
7. OSCC Biopsy specimens prior to neoadjuvant IT.
8. OSCC Resection specimens after anti-PD1 IT.

where 1 corresponds to the negative control, 2-4 represent inflammatory and potentially malignant oral diseases and 5-8 malignant oral diseases.

For this pilot study, Periodontitis tissue samples and Healthy Control Gingiva were harvested prospectively. The clinical study was approved by the Ethics Committee of the University of Erlangen-Nürnberg, Erlangen, Germany (approval number: 23-245_1-B) and performed in accordance with the Declaration of Helsinki. The other tissue samples were retrospectively selected and obtained from the Department of Pathology, University Hospital Erlangen. Ethical approval for OSCC Specimens (approval number: 17-54-Bp, Ethics Committee of the University of Erlangen-Nürnberg) as well as Oral Leukoplakia and Oral Lichen Planus (approval number: 16-282-B, Ethics Committee of the University of Erlangen-Nürnberg) was available.

### Patients collective

For the current pilot study, a tissue microarray (TMA) for multiplex immunhistological analysis was created and samples of a total of 31 patients were analyzed. The collective included healthy control gingiva of 5 patients after minor oral surgery procedures like third molar extractions without signs of acute infection. Periodontitis (PD) samples were obtained from 5 patients diagnosed with severe Periodontitis (Stage 4/Grade C (15); (CAL ≥ 5mm, loss of ≥ 5 teeth, smoking of ≥ 10 cigarettes/day, no diabetes) before the beginning of treatment (Department of Operative Dentistry and Periodontology, University Hospital Erlangen) and prior to tooth extractions (Oral- and Cranio-Maxillofacial Surgery, University Hospital Erlangen). Sufficient assessment of gingiva samples was only possible in 4 of the patients due to inaccuracies in TMA punching. Oral Leukoplakia (OL) samples from 4 patients and Oral Lichenoid Lesions from 4 additional individuals without histologic signs of dysplasia were included. All included patients with pathologic diagnosis of an Oral Lichenoid Lesion showed clinical signs of Oral Lichen Planus (OLP) and therefore the diagnosis of OLP was made. Additionally, tissue samples from 5 patients diagnosed with OSCC without lymphatic metastases (OSCC N0) and from 5 OSCC-patients with metastases (OSCC N+) were included. From the OSCC N+ group, only 4 patients were available for analysis as due to punching inaccuracies the tumor tissue was not sampled in one patient. OSCC biopsies prior to anti-PD1 immunotherapy (IT) and resection specimens after IT from 3 patients were also included. The patients received 1 dose of Pembrolizumab 200mg i.v. 14 days prior to tumor resection surgery as an individual healing attempt. Due to the described challenges during TMA construction, a total number of 29 patients were available for analysis. Samples of the control tissue (human tonsil and salivary gland) were obtained during routine tonsillectomy and submandibular glad removal due to benign pathologies and in absence of acute inflammation (no acute tonsillitis and sialadenitis).

### TMA creation

TMA containing 68 samples and six controls (human tonsil and salivary gland) was planned. For each target tissue (e.g. healthy control of Gingiva, patient 1), two individual tissue samples were intended. For this purpose, two regions of interest were selected on one or more digitalized HE-stained slides of the original FFPE. Regions were matched by images of Slides and corresponding FFPE blocks using G-TMA Software and afterwards TMA cores (diameter of 1.5mm) were performed automatically by the TMA Grand Master (G-TMA, Vers.2, 3D Histech, Budapest, Hungary).

### Immunofluorescence staining

The immunofluorescence protocol was performed using an autostainer (Leica Bond RX^m^, Leica Biosystems, CA, USA), which performs all steps of the staining procedure including pretreatment. The autostainer was loaded with the TMA slide and required reagents. In the initial stage of the pretreatment, the “Dewax” program, developed by Leica, was used. First, the Bond Dewax Solution (REF: AR9222, Leica Biosystems, Newcastle Upon Tyne, UK) was applied for 30 minutes at 72°C, to remove the paraffin from the tissue, followed by a brief treatment with isopropanol. Next the TMA was subjected to heat induced epitope retrieval in EDTA buffer (Bond Epitope Retrieval Solution 2, pH 9.0, Ref: AR9640, Leica Biosystems, Newcastle Upon Tyne, UK) at 95-100°C for 20 minutes to expose the target antigens and allow the primary antibody to bind. Non-specific binding sites were then blocked by incubation for 5 min in Antibody Diluent/Block Solution (REF: ARD1001EA, Akoya Biosciences, Marlborough, MA, USA). The first primary antibody, CD11c (Abcam, REF: AB52632, Cambridge, UK; dilution 1:1500 in 1x Antibody Diluent/Block) was then added and incubated for 30 min. For signal amplification, 1X Opal Anti-Ms + Rb HRP (Akoya Biosciences, Marlborough, MA, USA; REF: ARH1001EA) was used as a secondary antibody and Opal polymer and applied for 10 min. The Opal Anti-MS polymer binds to the CD11c antibody and creates a binding site for the first Opal 570 (dilution 1:150 in 1x Plus Automation Amplication Diluent, FP1609, Akoya Biosciences, Marlborough, USA), which was applied for 10 min in the next step. To apply the second primary antibody IL-23R (Abcam, REF: AB255964, Cambridge, UK; dilution 1:400), antigen retrieval was repeated, this time in citrate buffer pH 6.0 (Bond Epitope Retrieval Solution 1 REF: AR9961, Leica Biosystems, Newcastle Upon Tyne, UK) for 30 min. The blocking buffer was then incubated for 5 min and the second antibody IL-23R was applied for 30 min. The Opal Anti-MS polymer was incubated for 10 minutes and the second Opal 690 (1:150 dilution) was added in the same manner as the first primary antibody. For the application of the third antibody CD68 (1:3000, DAKO M0814, Santa Clara, CA, USA) the steps were repeated as for the second antibody. Antigen retrieval pretreatment was performed with EDTA pH9.0 for 20 minutes. This was followed by incubation with blocking buffer for 5 min, followed by incubation with CD68 antibody for 30 min. Signal amplification and fluorophore binding were performed as for the other two primary antibodies. Then Opal 520, also diluted 1:150 was applied and incubated for 10 min. The final primary antibody CD163 (Novocastra, REF: 6089068, Leica Biosystems Newcastle, UK) was applied after repeated citrate-based epitope retrieval and blocking of the non-specific binding sites for 5 min. After incubation of the CD163 antibody and the Opal polymer, a 10-minute incubation of the Opal-DIG reagent (included in the Opal 780 Reagent Pack, REF: FP1501001KT; Akoya Biosiences, Marlborough, USA) is required for the last Opal 780 to further label and amplify the signal. The last Opal 780, diluted 1:25 in Antibody Diluent/Block, was then added and incubated for 60 min. Finally, the TMA was incubated in Spectral DAPI for 10 min for nuclear staining. The TMA slide was then immersed briefly in distilled water and covered with antifade mounting medium (Vectashield, REF: H-1000, Vector Laboratories, Newark, CA, USA) and a coverslip. The TMA slide was stored in a cool, dark place until scanning.

### Digitalization of the specimens

**Figure 1:**
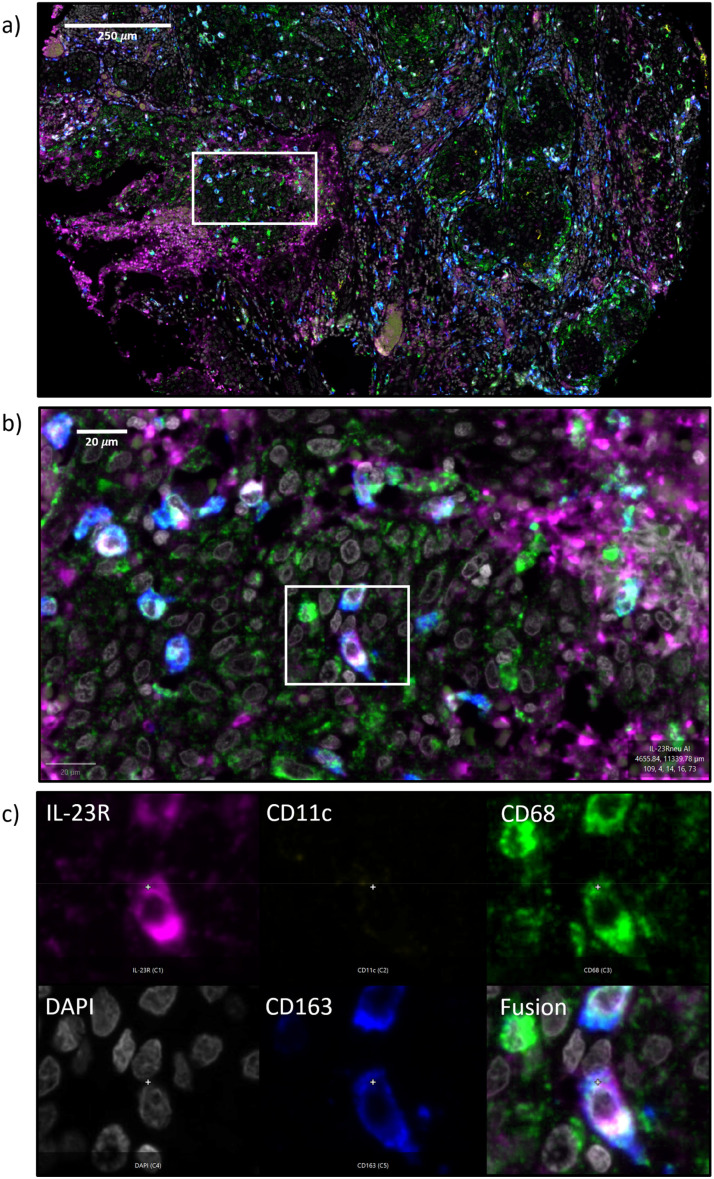
**(a–c)** Immunofluorescence 4x multi-staining The figure shows an exemplary micrograph of an oral squamous cell carcinoma (OSCC) tumor resection specimen (**Figure 1a**) and a high-power magnification (Fib. 1B). **Figure 1c** shows a magnification on single-cell level (200x magnification) and a separate image for each fluorescence channel: IL-23R (magenta), CD11c (yellow), CD68 (green), DAPI (white), CD163 (blue) and a fusion image.

The TMA slide was scanned with a Hamamatsu scanner (NanoZoomer S60, Shizuoka, Hamamatsu Photonics K.K., Japan) (**Figure 1**). ROI for scanning was selected, secondly, three focus points were defined for each TMA core. The following Opals were used for the staining described above (CD11c, IL-23R, CD68 and CD163). Opal 520, Opal 570, Opal 690 and Opal 780 (included in Opal 3-Plex Detection Kit and Opal 780 Reagent Pack), each Opal representing one of the primary antibodies used and is visible at a different wavelength. Opal 520 f.ex. was measured using the FITC color channel and stimulated at a wavelength lower than 520 nm. Accordingly, Opal 570 was measured in the TxRed channel, Opal 690 in Cy5 and Opal 780 was detected in the Cy7 channel.

In practice, the required filters were configured under the “Review Fluo” settings. Setup was started with the DAPI channel. The next step is to optimize the “scaling”, which represents the stretching or compression of the brightness values in order to use the entire dynamic range. Exposure time for DAPI was 40ms and a scaling of 1x was selected. The FITC channel, to detect the CD68 antibody, was set with an exposure time of 50ms and a scaling value of 1x. For this and the following color channels, an “offset” was also set, to correct the background values to avoid background noise. For the Opal 570, the TxRed channel was used with an exposure time of 70ms and a scaling of 1x. For the fourth color channel Cy5, which measures Opal 690 to detect the IL23R antibody, an exposure time of 120ms and a scaling of 2x was selected. The last color channel Cy7 was configured with an exposure time of 150ms and a scaling of 2x. Once all channels have been processed, the individual focus points were optimized using the “Review Focus” function to obtain a sharp overall image. Reviewing was performed in NDP.view2 (Shizuoka, Hamamatsu Photonics K.K., Japan).

### Cell counting

The software QuPath v0.5.1 (16) was used for analysis and quantitative cell density assessment. The whole slide image of the digital fluorescence TMA slide was loaded in QuPath as.ndpis file. Each TMA segment was manually selected. Missing tissue as well as visible artefacts were manually removed from the area for cell counting. Thus, a low level of autofluorescence through erythrocytes as well as an accurate selection of the tissue should be achieved. In each sample, a cell detection operation using the DAPI channel was performed. The intensity threshold was selected (value 5) and cell expansion was set (3 µm; including cell nucleus). The other parameters were set as standard (Requested pixel size: 0,5 µm; Background radius: 8 µm; Median filter radius 0 µm; Sigma 1.5 µm; minimum area 10 µm ^2^; maximum area 400 µm ^2^). Thus, the total cell count for each specimen was obtained. Successful cell detection was also manually confirmed. For identification of IL-23R, CD11c, CD68 and CD163 positive cells, individual object classifier were trained. For this purpose, the Artificial neural network classifier ANN_MLP was used. Then, for each fluorescence channel, positive and negative cells were marked manually and used for neural net training. This process was monitored using the “live review” feature in the whole specimen. Training was stopped after positive and negative cells were identified by the neural net in a similar way the investigator (LT) would count these cells manually (following a supervied learning approach). This was performed individually for IL-23R, CD11c, CD68 and CD163. Next, the trained classifiers were tested on different tissue types and the results were compared with manual cell counting of the investigators (LT, MW). As a sufficient cell detection was noticed, the described cell detection based on DAPI and the trained classifiers for IL-23R, CD11c, CD68 and CD163 were used for all samples. Data were exported in an Excel file and processed further.

### Statistical analysis

For each patient sample, two TMA punches were available. For subsequent calculation, the mean value of the total cell count and of each marker was calculated from both punches. For each marker, the number of positive cells per mm^2^ specimen area and the Labeling Index (LI) as percentage of positive cells versus all cells were calculated. In addition, the expression ratios of CD163 positive cells vs. CD11c positive cells as well as CD163 positive cells vs. CD68 positive cells were calculated in Microsoft Excel. Statistical analysis was then performed with SPSS Version 28.0. Initially, normal-distribution was tested using the Shapiro-Wilk test and the Kolmogorov-Smirnov test. It was shown that not all parameters showed normal-distribution. Therefore, the Mann-Whitney-U test was used to test for statistic significant differences.

## Results

### TMA creation and immunofluorescence multi-staining

The TMA was created successfully. In total, the TMA was planned to contain 68 tissue samples and six samples with staining quality controls. Out of the 68 samples on the TMA, 10 were not suitable for cell counting after immunofluorescence staining. In two of these, the tumor tissue in a N+ OSCC case was not hit. Therefore, the intended number of valid cases in this group was reduced from 5 to 4 cases. The other 8 TMA punches that were not used for cell counting either showed no epithelia cells or contained only tissue fragments. Due to the duplicates used in the TMA for each case, this did not lead to loss of another case. We therefore consider the approach for TMA creation described here as suitable for tissue characterization, even if the number of available cases is reduced by punching or precision`s limitations (as described above).

**Figure 2:**
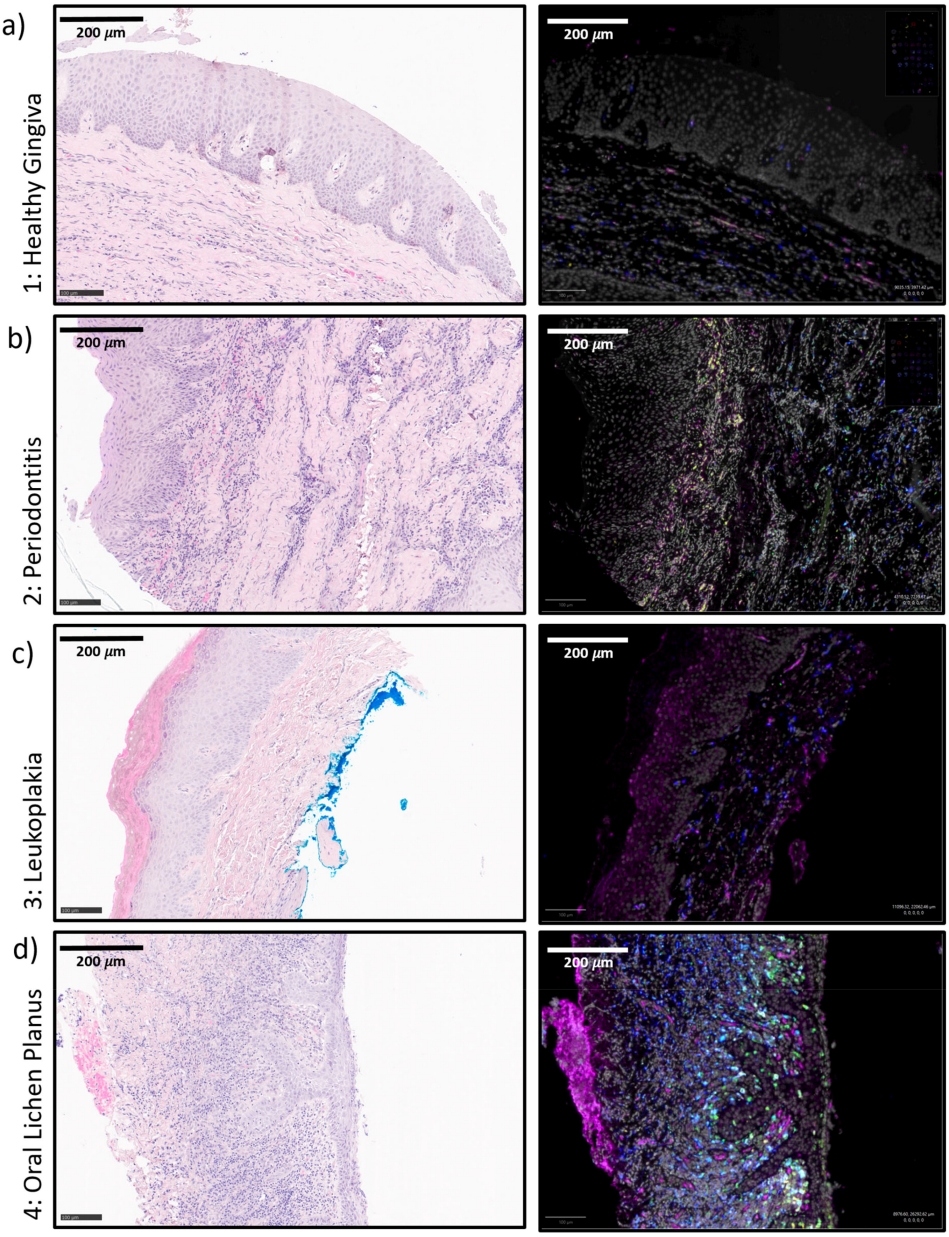
**(a–d)** Exemplary tissue micrographs (1-4) This figure shows exemplary micrographs for each analyzed tissue. On the left column, HE-stainings are displayed. On the right column, the corresponding immunofluorescence stainings are given. The immunofluorescence fusion images show DAPI (white), IL-23R (magenta), CD11c (yellow), CD68 (green) and CD163 (blue). **(a)** 1: Healthy Control Gingiva **(b)** 2: Periodontitis **(c)** 3: Oral Leukoplakia **(d)** 4: Oral Lichen Planus.

**Figure 3:**
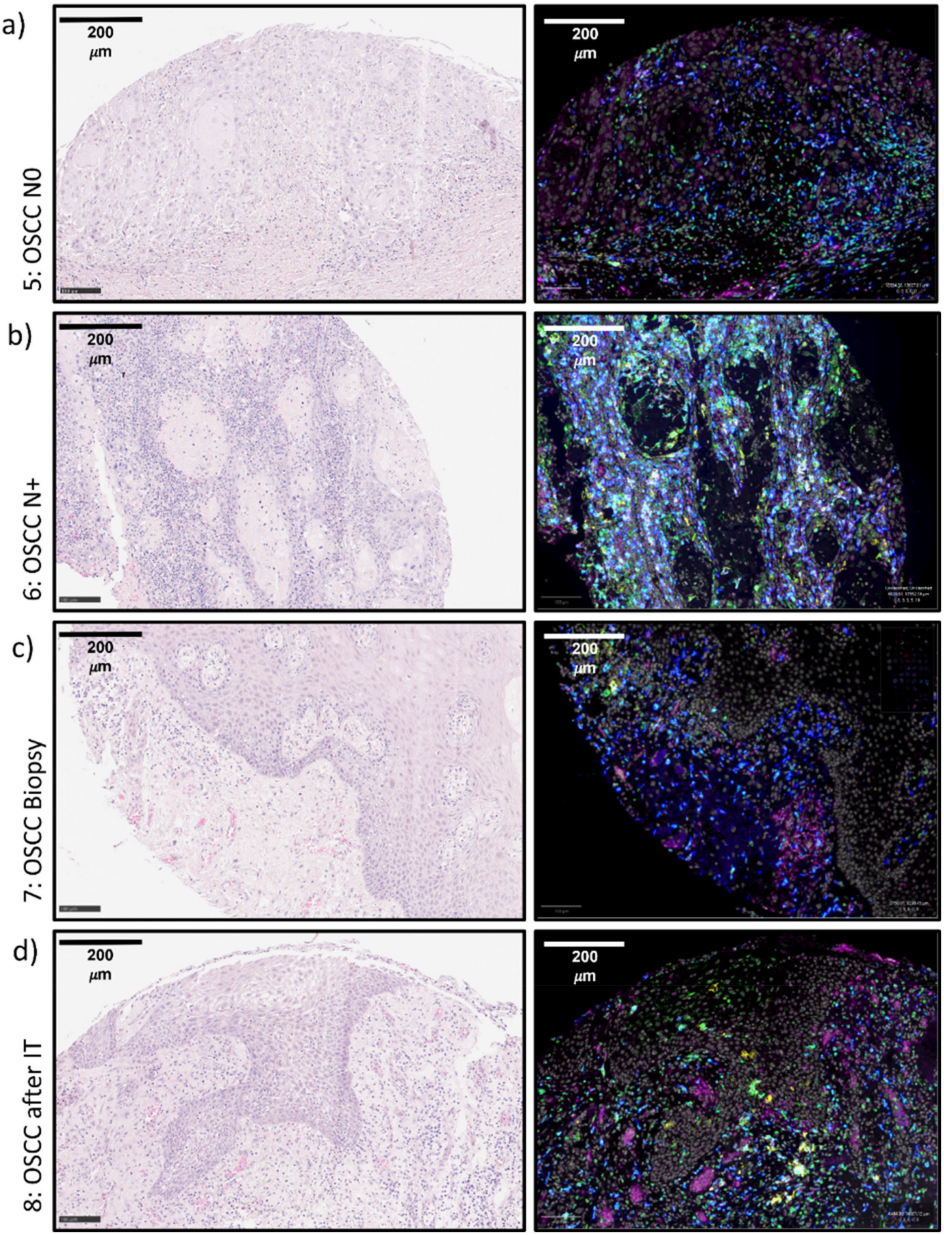
**(a–d)** Exemplary tissue micrographs (5-8) This figure shows exemplary micrographs for each analyzed tissue. On the left column, HE-stainings are displayed. On the right column, the corresponding immunofluorescence stainings are given. The immunofluorescence fusion images show DAPI (white), IL-23R (magenta), CD11c (yellow), CD68 (green) and CD163 (blue). **(a)** 5: OSCC N0 **(b)** 6: OSCC N+ **(c)** 7: OSCC Biopsy **(d)** 8: OSCC Resection after IT *OSCC, oral squamous cell carcinoma; IT, Immunotherapy with anti-PD1 antibodies*.

The fluorescence staining and the scanning process of the TMA was successfully and the digital slide of it could be captured in one attempt and saved as a single file (**Figures 2**, **3**).

### Oral inflammatory and benign diseases with potential for malignant transformation

**Figure 4:**
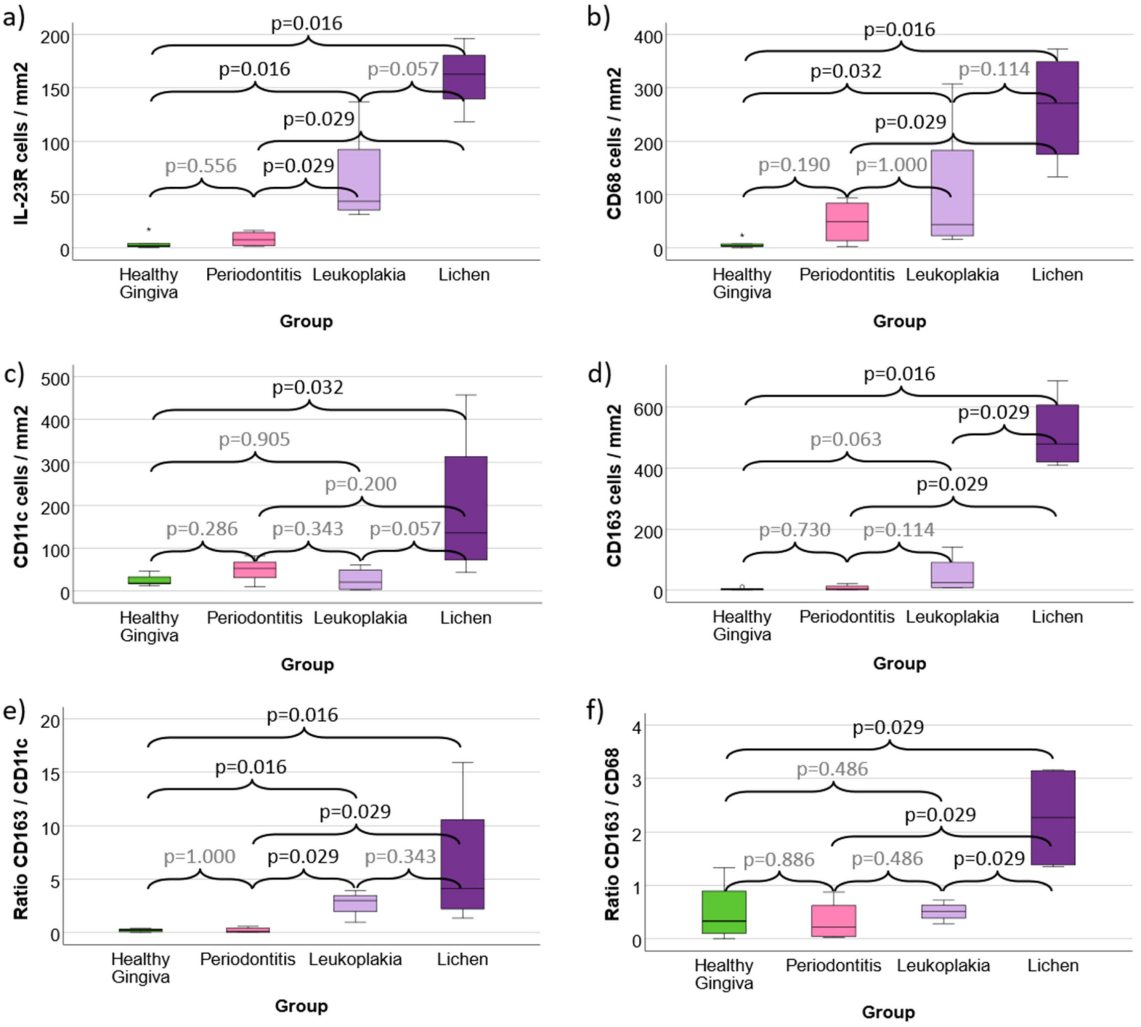
**(a–f)** Cell density and expression ratios in Periodontitis, oral Leukoplakia, oral Lichen Planus and healthy Gingiva controls **(a)** Cell density of IL-23 Receptor expressing cells **(b)** Cell density of CD68 positive macrophages **(c)** Cell density of CD11c positive macrophages (predominantly M1-like) and dendritic cells **(d)** Cell density of CD163 positive macrophages (predominantly M2-like) **(e)** CD163 / CD11c expression ratio as indication of M2 polarization **(f)** CD163 / CD68 expression ratio as indication of M2 polarization Values for Healthy Gingiva, Periodontitis, oral Leukoplakia and oral Lichen Planus are given. **Figures 4a-d** shows the cell density (cells/mm^2^ specimen area) of IL-23R, CD68, CD11c and CD163 positive cells. **Figures 4e, f** give the rations of all CD163 expressing cells versus all CD11c and CD68 expressing cells as indicator of M2 polarization of macrophages in the analyzed tissues. p-values generated using the Mann-Whitney U test are shown.

Comparing Periodontitis samples with Healthy Gingiva Controls, no significant expression difference of the analyzed markers was found (**Tables 1**, **2**, **Figure 4**). However, the mean value of CD68 and CD11c positive cells (49 and 49 cells/mm^2^) showed higher values compared to controls (8 and 25 cells/mm^2^) (**Table 1**).

**Table 1:** Cell density (cells/mm^2^) and expression ratios.

Group	Marker (mean, median and SD)					
	IL-23R cells/mm^2^	CD68 cells/mm^2^	CD11c cells/mm^2^	CD163 cells/mm^2^	Ratio CD163/ CD11c	Ratio CD163/ CD68
1: Healthy Gingiva Controls (n=5)	5	8	25	4	0.22	0.50
	2	4	17	4	0.25	0.33
	7	10	14	4	0.17	0.59
2: Periodontitis (PD) (n=4)	8	49	49	8	0.23	0.33
	8	49	53	4	0.15	0.22
	8	42	30	10	0.26	0.39
3: Oral Leukoplakia (OL) (n=4)	64	103	26	50	2.73	0.51
	44	44	21	26	3.00	0.51
	49	137	28	63	1.26	0.18
4: Oral Lichen Planus (OLP) (n=4)	160	262	193	514	6.39	2.26
	163	271	135	480	4.15	2.27
	32	108	184	126	6.54	1.02
5: OSCC Resection N0 (n=5)	127	425	37	83	3.35	0.21
	97	447	31	79	4.07	0.16
	112	209	26	45	2.07	0.09
6: OSCC Resection N+ (n=4)	192	937	242	330	2.78	0.42
	199	890	179	258	1.17	0.24
	123	278	229	210	3.40	0.39
7: OSCC Biopsy (n=3)	132	649	52	190	3.87	0.26
	127	601	45	101	4.61	0.24
	69	261	24	175	1.48	0.15
8: OSCC Resection after IT (n=3)	486	1645	1091	1204	1.27	0.73
	369	1256	552	1272	0.99	0.66
	368	1417	1045	926	0.91	0.25
p-values						
1 vs. 2	0.556	0.190	0.286	0.730	1.000	0.886
1 vs. 3	**0.016**	**0.032**	0.905	0.063	**0.016**	0.486
1 vs. 4	**0.016**	**0.016**	**0.032**	**0.016**	**0.016**	**0.029**
1 vs. 5	**0.008**	**0.008**	0.690	**0.008**	**0.032**	0.556
1 vs. 6	**0.016**	**0.016**	**0.032**	**0.016**	**0.016**	0.886
1 vs. 7	**0.036**	**0.036**	0.250	**0.036**	**0.036**	0.857
1 vs. 8	**0.036**	**0.036**	**0.036**	**0.036**	**0.036**	0.400
2 vs. 3	**0.029**	1.000	0.343	0.114	**0.029**	0.486
2 vs. 4	**0.029**	**0.029**	0.200	**0.029**	**0.029**	**0.029**
3 vs. 4	0.057	0.114	0.057	**0.029**	0.343	**0.029**
5 vs. 6	0.730	**0.032**	0.063	**0.016**	0.730	0.286
7 vs. 8	0.057	0.114	0.057	**0.029**	0.343	**0.029**

The table shows the detected cell density (cells/mm^2^) in the different analyzed Groups of tissues (1-8) for IL-23R, CD68, CD11c and CD163 positive cells. In addition, the CD163 vs. CD11c and the CD163 vs. CD68 expression ratios are given. Values represent the median, mean and standard deviation (SD). Below, the p-values between different Groups (1-8) provided by the are Mann-Whitney U test are given. Bold denotes statistical significance (p < 0.05).

SD, Standard deviation; IT, immunotherapy with anti-PD1, OSCC, oral squamous cell carcinoma; n, number of cases.

**Table 2:** Labeling Indices (LI).

Group	Marker (mean, median and SD)			
	IL-23R LI	CD68 LI	CD11c LI	CD163 LI
1: Healthy Gingiva Controls (n=5)	0.14	0.22	0.63	0.12
	0.09	0.08	0.75	0.11
	0.18	0.25	0.24	0.11
2: Periodontitis (PD) (n=4)	0.18	0.80	0.97	0.14
	0.13	0.80	1.10	0.06
	0.19	0.66	0.63	0.18
3: Oral Leukoplakia (OL) (n=4)	2.20	3.81	0.92	1.86
	1.16	1.26	0.60	0.75
	2.33	5.70	1.09	2.62
4: Oral Lichen Planus (OLP) (n=4)	3.40	4.99	3.06	10.43
	3.95	4.44	2.81	10.13
	1.34	2.31	1.95	4.78
5: OSCC Resection N0 (n=5)	2.16	7.27	0.64	1.42
	1.91	6.97	0.60	1.43
	1.58	3.55	0.41	0.74
6: OSCC Resection N+ (n=4)	3.79	16.63	4.96	6.44
	3.14	14.15	2.50	4.46
	2.70	6.53	6.10	5.72
7: OSCC Biopsy (n=3)	2.80	12.88	1.10	3.58
	3.26	11.99	1.28	2.93
	1.28	2.02	0.41	2.48
8: OSCC Resection after IT (n=3)	5.28	17.42	11.70	13.61
	5.71	19.39	8.56	16.43
	1.99	8.78	6.03	7.83
p-values				
1 vs. 2	0.730	0.190	0.413	0.905
1 vs. 3	**0.016**	**0.032**	1.000	0.063
1 vs. 4	**0.016**	**0.016**	**0.016**	**0.016**
1 vs. 5	**0.008**	**0.008**	1.000	**0.008**
1 vs. 6	**0.016**	**0.016**	**0.032**	**0.016**
1 vs. 7	**0.036**	**0.036**	0.250	**0.036**
1 vs. 8	**0.036**	**0.036**	**0.036**	**0.036**
2 vs. 3	**0.029**	0.486	0.686	0.114
2 vs. 4	**0.029**	**0.029**	0.114	**0.029**
3 vs. 4	0.343	0.343	0.200	0.057
5 vs. 6	0.413	**0.016**	**0.032**	**0.032**
7 vs. 8	0.343	0.343	0.200	0.057

The table shows Labeling Indices (LI) in the different analyzed Groups of tissues (1-8) for IL-23R, CD68, CD11c and CD163 positive cells. The LI represents the number of positively counted cells for each marker divided through the total cell count (DAPI positive nuclei). Values represent the median, mean and standard deviation (SD). Below, the p-values between different Groups (1-8) provided by the are Mann-Whitney U test are given. Bold denotes statistical significance (p < 0.05).

SD, Standard deviation; IT, immunotherapy with anti-PD1; OSCC, oral squamous cell carcinoma; n, number of cases.

In contrast, Oral Leukoplakia samples showed a significantly (p ≤ 0.032) increased cell density and Labeling Index (LI) of IL-23R and CD68 positive cells compared to Controls (mean IL-23R 64 cells/mm^2^ vs. 5 cells/mm^2^ and mean CD68 103 cells/mm^2^ vs. 8 cells/mm^2^) (**Tables 1**, **2**, **Figure 4**). In addition, the CD163/CD11c ratio in Oral Leukoplakia was significantly (p=0.016) increased (**Table 1**, **Figure 4**).

Similar values were found comparing Oral Lichen Planus with Healthy Gingiva Controls. The cell density and Labeling Index of all analyzed markers (IL-23R, CD68, CD11c and CD163) were significantly (p ≤ 0.032) higher compared to Healthy Controls (see **Tables 1**, **2**, **Figure 4**). In addition, the CD163/CD11c and the CD163/CD68 expression ratios were significantly (p ≤ 0.029) higher in Oral Lichen compared to Controls indicating a shift towards M2 polarization of macrophages (6.39 and 2.26 vs. 0.22 and 0.50) (**Table 1**, **Figure 4**).

Comparing Oral Leukoplakia and Oral Lichen with Periodontitis, both potentially malignant transforming lesions showed significantly (p=0.029) increased IL-23R cell densities and Labeling Indices as well as a significantly increased CD163/CD11c ratio (p=0.029) (see **Tables 1**, **2**, **Figure 4**). Additionally, Oral Lichen Planus showed a significantly (p=0.029) increased CD68 and CD163 cell density and Labeling Index (see **Tables 1**, **2**, **Figure 4**).

Comparing Oral Lichen Planus and Oral Leukoplakia, CD163 cell density (mean 514 cells/mm^2^ vs. 50 cells/mm^2^, p=0.029) and CD163/CD68 expression ratio (2.26 vs. 0.51, p=0.029) were significantly higher in Lichen lesions (**Table 1**, **Figure 4**).

### Oral squamous cell carcinomas with and without lymph node metastases (N+ vs. N0)

**Figure 5:**
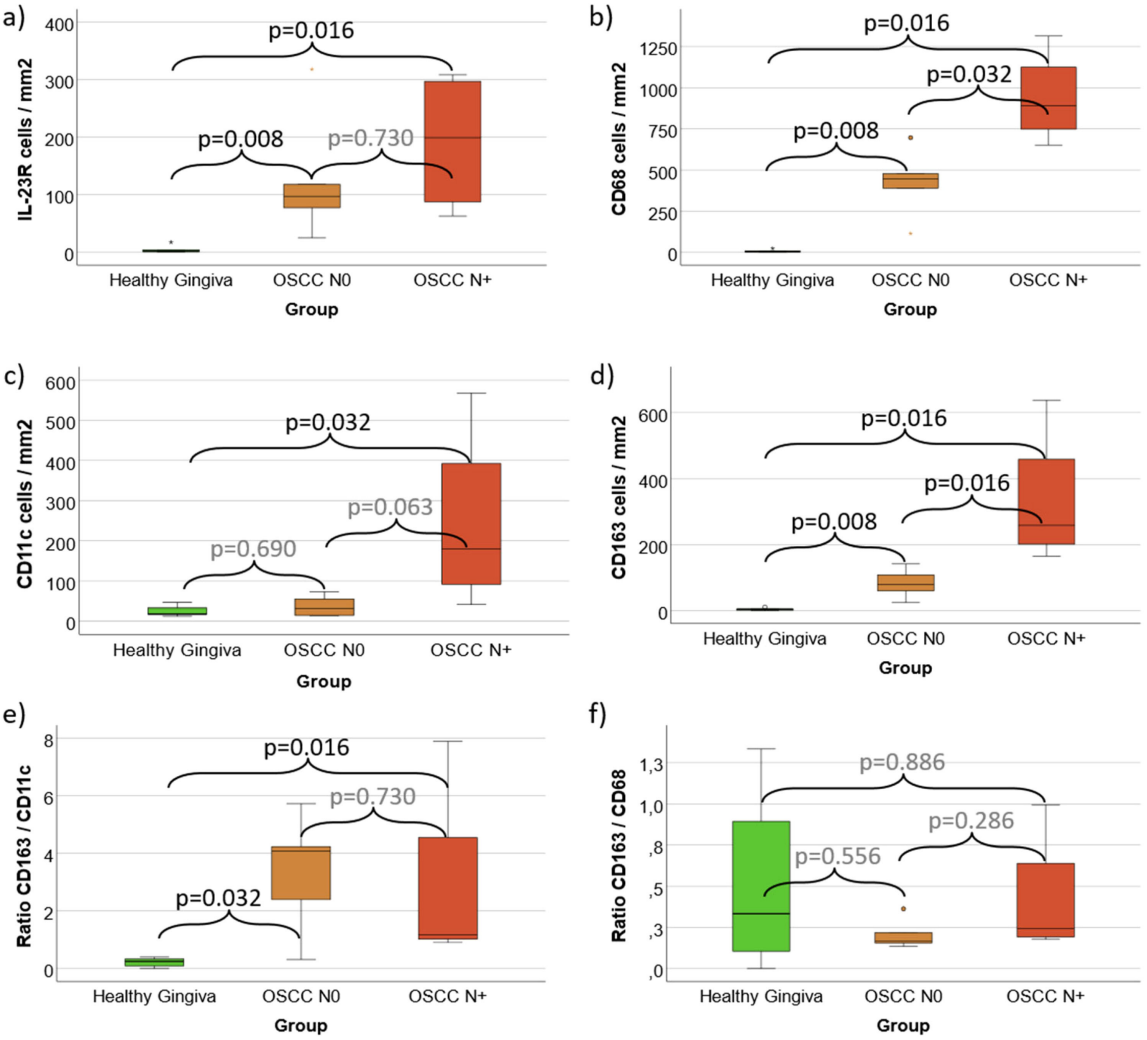
**(a–f)** Cell density and expression ratios in N0 OSCC and N+ OSCC compared with controls **(a)** Cell density of IL-23 Receptor expressing cells **(b)** Cell density of CD68 positive macrophages **(c)** Cell density of CD11c positive macrophages (predominantly M1-like) and dendritic cells **(d)** Cell density of CD163 positive macrophages (predominantly M2-like) **(e)** CD163 / CD11c expression ratio as indication of M2 polarization **(f)** CD163 / CD68 expression ratio as indication of M2 polarization Values for Healthy Gingiva, oral squamous cell carcinoma (OSCC) resection specimens without lymph node metastases (N0) and cases with lymph node metastases (N+) are given. **Figures 5a-d** shows the cell density (cells/mm^2^ specimen area) of IL-23R, CD68, CD11c and CD163 positive cells. **Figures 5e, f** give the rations of all CD163 expressing cells versus all CD11c and CD68 expressing cells as indicator of M2 polarization of macrophages in the analyzed tissues. p-values generated using the Mann-Whitney U test are shown.

Compared to healthy Gingiva controls, OSCC tumor resection specimens without (N0) and with (N+) lymph node metastases showed a significantly increased infiltration of IL-23R expressing cells (mean 5 cells/mm^2^ vs. 127 cells/mm^2^ and 192 cells/mm^2^; p ≤ 0.016) (**Table 1**, **Figure 5a**). The difference between N0 and N+ cases was not statistically significant. Corresponding values were seen when assessing the LI (**Table 2**).

CD68 cell density in healthy controls (mean 8 cells/mm^2^) was significantly (p ≤ 0.016) lower than in N0 OSCC (mean 425 cells/mm^2^) and N+ OSCC (mean 937 cells/mm^2^) cases (**Table 1**, **Figure 5b**). The difference between N0 and N+ was also statistically significant (p=0.032).

Healthy Gingiva showed a significantly (p=0.032) lower CD11c cell density (mean 25 cells/mm^2^) than N+ OSCC specimens (mean 242 cells/mm^2^) (**Table 1**, **Figure 5c**). The difference between Controls and N0 OSCC as well as between N0 and N+ OSCC was not statistically significant (**Table 1**, **Figure 5c**).

CD163 cell density in healthy controls (mean 4 cells/mm^2^) was significantly (p ≤ 0.016) lower than in N0 OSCC (mean 83 cells/mm^2^) and N+ OSCC (mean 330 cells/mm^2^) cases (**Table 1**, **Figure 5c**). The difference between N0 and N+ was also statistically significant (p=0.016). Similar expression differences were found for the LI of CD68, CD11c and CD163 that are shown in **Table 2**.

The CD163/CD11c expression ratio in N0 OSCC (mean 3.35) and N+ OSCC (mean 2.78) was significantly (p ≤ 0.032) higher compared to Controls (mean 0.22) (**Table 1**, **Figure 5d**). There was no significant difference between N0 and N+ OSCC cases.

The CD163/CD68 expression ratio showed no significant expression differences between Controls, N0 and N+ cases (**Table 1**, **Figure 5e**).

### Oral squamous cell carcinomas before and after anti-PD1 Immunotherapy

**Figure 6:**
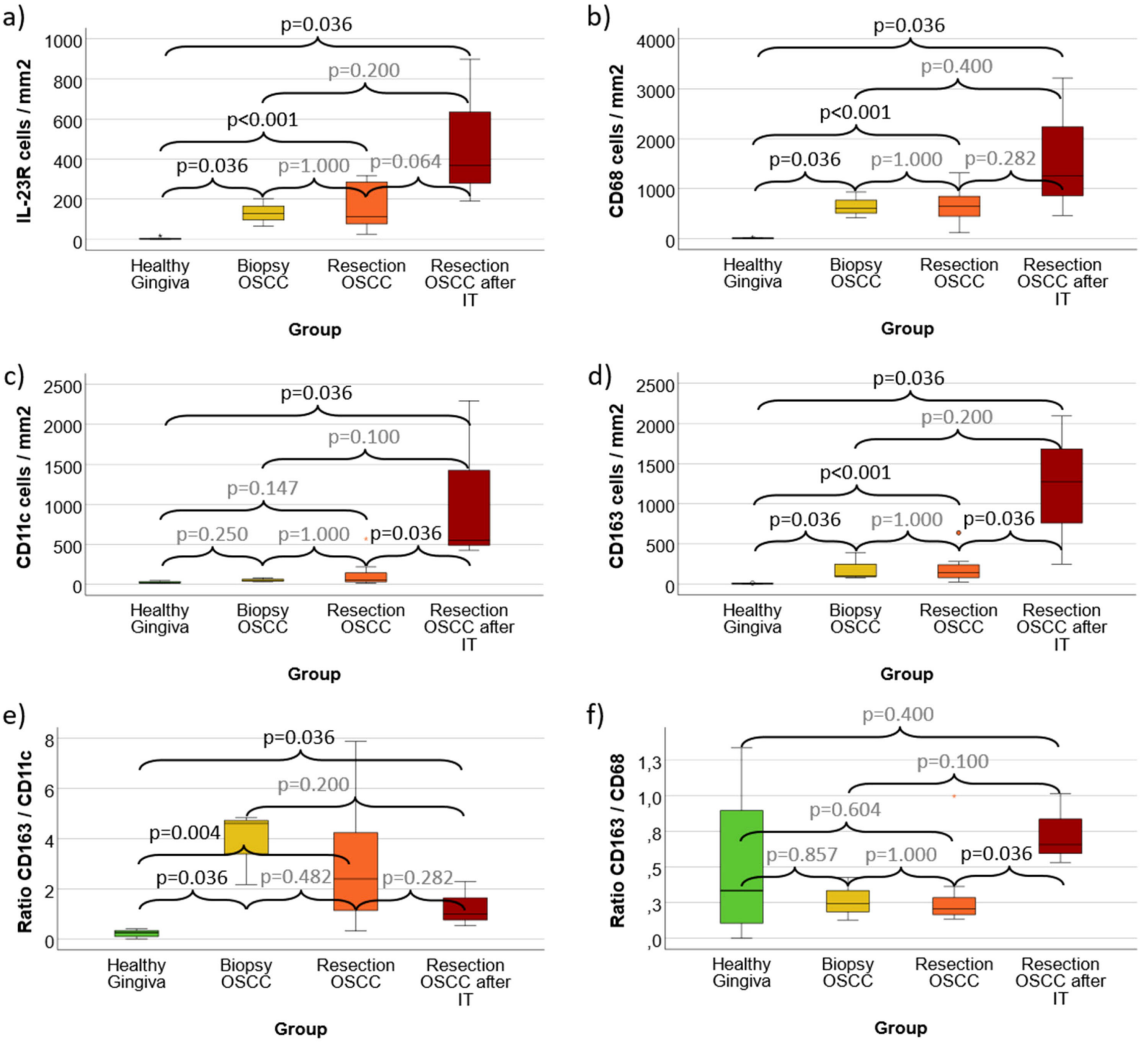
**(a–f)** Cell density and expression ratios in OSCC Biopsies, Resection specimens and Resection specimens after anti-PD1 immunotherapy (IT) compared with controls **(a)** Cell density of IL-23 Receptor expressing cells **(b)** Cell density of CD68 positive macrophages **(c)** Cell density of CD11c positive macrophages (predominantly M1-like) and dendritic cells **(d)** Cell density of CD163 positive macrophages (predominantly M2-like) **(e)** CD163 / CD11c expression ratio as indication of M2 polarization **(f)** CD163 / CD68 expression ratio as indication of M2 polarization Values for Healthy Gingiva, oral squamous cell carcinoma (OSCC) Biopsy specimens, OSCC tumor resection samples (irrespective of the N-Status) and tumor resection specimens after anti-PD1 immunotherapy (IT) are given. Biopsy samples and specimens after IT were obtained from the same patients. **Figures 6a-d** shows the cell density (cells/mm^2^ specimen area) of IL-23R, CD68, CD11c and CD163 positive cells. **Figures 6e, f** give the rations of all CD163 expressing cells versus all CD11c and CD68 expressing cells as indicator of M2 polarization of macrophages in the analyzed tissues. p-values generated using the Mann-Whitney U test are shown.

In addition to the aforementioned parameters, a comparison between Healthy Gingiva, OSCC Biopsy specimens prior to anti-PD1 immunotherapy (IT) and OSCC Resection specimens after IT as well as OSCC Resection specimens that did not receive any IT was performed. For the last group, the OSCC N0 and N+ Resection specimens (Groups 5 and 6 in **Table 1**) were pooled. The results are shown in **Figure 6**.

IL-23R expression in all analyzed malignant groups (OSCC Biopsy, OSCC Resection and OSCC Resection after IT) was significantly (p ≤ 0.036) higher compared to Healthy Gingiva Controls (mean 5 cells/mm^2^, 132 cells/mm^2^, 156 cells/mm^2^ and 486 cells/mm^2^) (**Figure 6a**). There was no significant difference between biopsies and tumor resection specimens as well as specimens with and without IT (**Figure 6a**).

CD68 cell density in all analyzed malignant groups (OSCC Biopsy, OSCC Resection and OSCC Resection after IT) was significantly (p ≤ 0.036) higher compared to Healthy Gingiva Controls (mean 8 cells/mm^2^, 649 cells/mm^2^, 653 cells/mm^2^ and 1645 cells/mm^2^) (**Figure 6b**). There was no significant difference between biopsies and tumor resection specimens as well as specimens with and without IT (**Figure 6b**).

Tumor Resection specimens after IT (mean 1091 cells/mm^2^) showed a significantly (p=0.036) increased CD11c cell density compared to Heathy Gingiva Controls (mean 25 cells/mm^2^) as well as compared to OSCC Resection specimens without IT (mean 128 cells/mm^2^) (**Figure 6c**). Other differences of CD11c expression were not statistically significant (**Figure 6C**).

CD163 cell density in Tumor Resection specimens after IT (mean 1204 cells/mm^2^) was significantly (p=0.036) higher compared to Heathy Gingiva Controls (mean 4 cells/mm^2^) as well as compared to OSCC Resection specimens without IT (mean 192 cells/mm^2^) (**Figure 6d**). In addition, OSCC Biopsy samples (mean 190 cells/mm^2^) as well as Resection specimens (without IT) showed significantly (p ≤ 0.036) higher CD163 expression compared to Controls (**Figure 6d**).

The CD163/CD11c expression ration in OSCC Biopsy Specimens (mean 3.87), in OSCC Resection samples without IT (mean 3.10) and in Tumor Resections after IT (mean 1.27) was each significantly (p ≤ 0.036) higher compared to Healthy Controls (mean 0.22) (**Figure 6d**). Other differences in the CD163/CD68 expression ratio were not statistically significant (**Figure 6d**).

OSCC Tumor Resection specimens after IT showed a significantly higher CD163/CD11c expression ratio compared to OSCC Resections without IT (mean 0.73 vs. 0.30; p=0.036) (**Figure 6e**). There was no significant difference between the other groups in CD163/CD11c expression ratio.

### Lineage of IL-23R expressing cells

**Figure 7:**
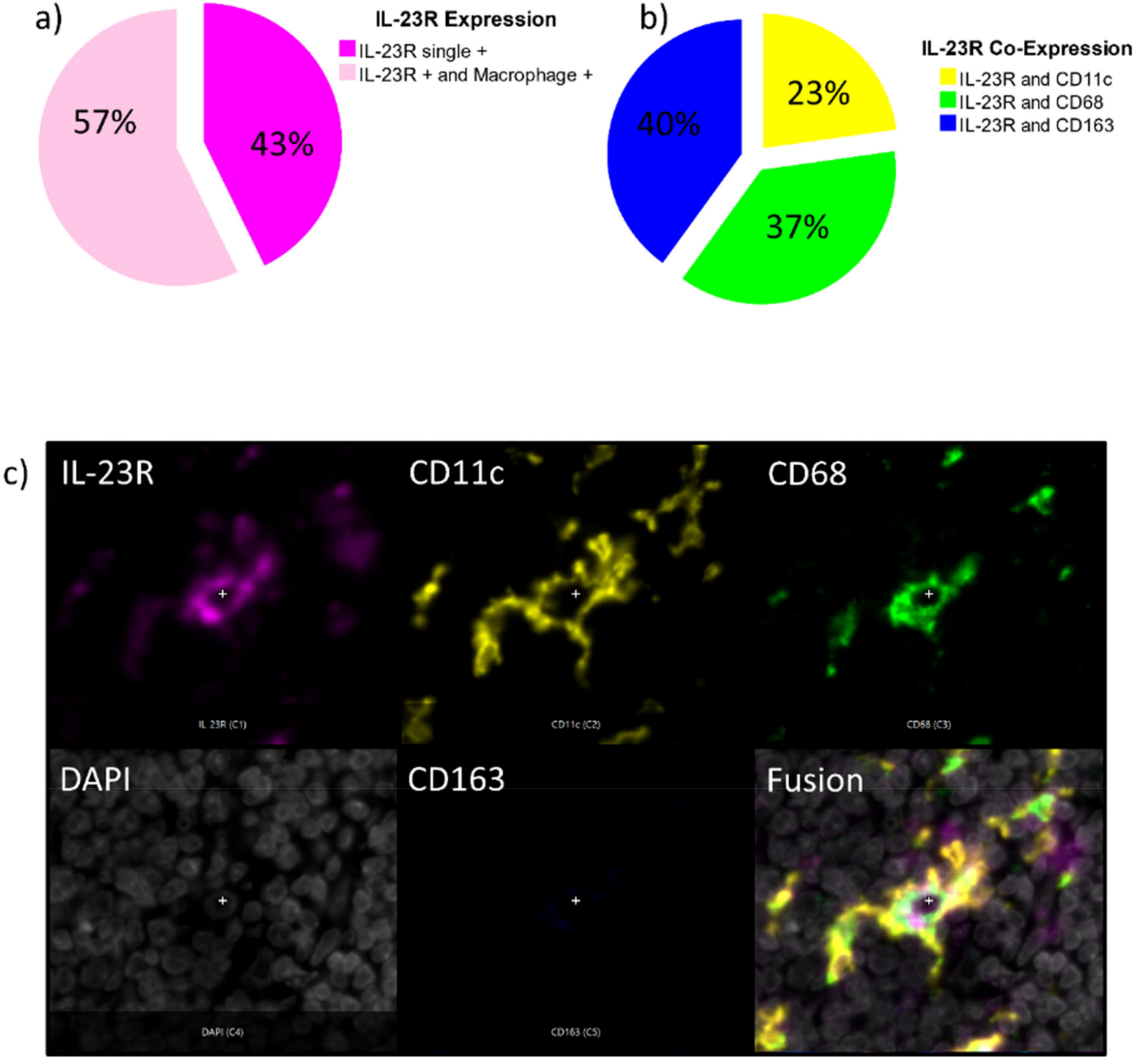
**(a–c)** Lineage of IL-23R expressing cells **Figure 7a** shows the percentage of IL-23R single positive cells compared to the Il-23R positive cells that co-express macrophage and Dendritic Cell markers (CD68, CD163, CD11c) in all analyzed tissue specimens. **Figure 7b** differentiates the IL-23 co-expressing cells in IL-23R and CD11c positive cells (yellow), IL-23R and CD68 positive cells (blue) as well as IL-23R and CD163 positive cells (blue). **Figure 7c** shows an exemplary magnification on single-cell level (200x) showing a CD11c+ CD68+ IL-23+ Dendritic Cell with typical morphology. A separate image for each fluorescence channel IL-23R (magenta), CD11c (yellow), CD68 (green), DAPI (white), CD163 (blue) and a fusion image are given.

All detected IL-23R positive cells in the current study were checked for co-expression of the macrophage and dendritic cell markers (CD68, CD11c and CD163) to further elucidate the lineage of IL-23R expressing cells. This analysis over all positive cells revealed that 43% of the IL-23R expressing cells were single-positive and 57% of the cells co-expressed CD68, CD11c, CD163 or several of these markers (**Figure 7a**). Of the IL-23R co-expressing cells, 23% were CD11c positive, 37% were CD68 positive and 40% showed to be CD163 positive (**Figure 7b**). There were also IL-23R positive cells that co-expressed multiple macrophage markers. **Figure 1c** shows cells co-expressing IL-23R, CD68 and CD163, which can be considered to be M2-like macrophages. In addition, there were also IL-23R, CD11c and CD68 positive cells comparable to the example shown in **Figure 7c**, that display Dendritic Cell morphology. In addition, there was a low number of quadruple positive cells detected (IL-23R, CD68, CD11c and CD163).

## Discussion

The current analysis did not reveal significant differences in IL-23R and macrophage marker expression between healthy Controls and Periodontitis (PD) samples. However, there was a tendency towards higher CD68 infiltration levels in PD which should be further investigated in a larger patient cohort. A previous study on macrophage polarization in human PD samples did show a significant increase in CD68 positive macrophages in PD compared to healthy Controls (17). However, there was no significant difference in iNOS positive M1-like macrophages and CD206 expressing M2-like macrophages between the analyzed groups. Interestingly, Gingivitis samples showed the highest expression levels of all three analyzed macrophage markers (17). These data indicate that acute inflammatory responses as they might be present in Gingivitis seem to generate the highest infiltration of macrophages. The chronic disease of PD might therefore show lower inflammatory infiltrate compared to an acute disease like Gingivitis.

The receptor of IL-23, IL-23R was identified in 2002 and has been mostly studied in the context of Th17 cells as Th17 inducing cytokine in mouse models (9). IL-23 supports the expansion and survival of established murine Th17 cells. However, conventional T-cells in the periphery showed IL-23R expression only upon activation (9). Additionally, IL-23R is expressed in NK cells and it was shown that IL-23R positive NK cells enrich in the synovial fluid in human spondylarthritis (9). In addition, auto-aggressive T-cells were shown to require IL-23R signaling to maintain their phenotype (18). Very little is known about IL-23R expression and function in myeloid cells. Expression of IL-23R was described in human macrophages, which responded to IL-23 treatment with increased secretion of proinflammatory cytokines (9). Additionally, activated granulocytes have been shown to express IL-23R in inflammatory murine models of colitis and aspergillosis. However, further characterization of IL-23R expression in human myeloid cells is still lacking.

In periodontal disease, IL-23R expression is upregulated on various immune cells, including T-cells and myeloid cells, contributing to the expansion of the Th17 response. The interaction between IL-23 and IL-23R enhances the inflammatory response in periodontal tissues. Elevated levels of IL-23 and its receptor have been linked to increased Th17 cell activity and the subsequent production of IL-17 that contributes to the recruitment of neutrophils and other inflammatory cells to the site of infection. It also stimulates the production of other proinflammatory mediators, such as IL-6, TNF-α, and prostaglandins, which can lead to tissue destruction and bone resorption, characteristic of periodontitis. The IL-23/IL-17 axis is crucial in periodontal disease. IL-23 activates Th17 cells, which in turn produce IL-17. This creates a feedback loop that sustains chronic inflammation in periodontal tissues. This chronic inflammation and imbalance in immune regulation are thought to drive the progression of periodontal disease, leading to the destruction of the periodontal ligament and alveolar bone (19, 20). Although IL-23 is mostly described as cytokine activating Th17 cells, in our study 57% of IL-23R expressing cells co-expressed a macrophage marker. In a mouse model, IL-23 signaling led to a distinct macrophage subpopulation that could be differentiated from M1 or M2 polarized macrophages (21). These IL-23 activated macrophages produced IL-17A, IL-22 and interferon gamma (IFN-γ) analog to Th17 T-cells in a RORγT dependent manner. The IL-23 treated macrophages significantly promoted dermatitis in a psoriasis-like disease mouse model (21). This could indicate that IL-23R expressing macrophages could also show different functional properties in inflammatory and malignant diseases of the oral cavity.

Oral Leukoplakia (OL) showed a significant increase in macrophage infiltration as well as M2 polarization compared to healthy Controls in the current analysis. This shift towards macrophage infiltration as well as M2 polarization was even stronger in oral cancer samples (OSCC). These data indicate that cellular changes during the malignant transformation of oral epithelium are accompanied by an increased macrophage density and shift towards M2. A previous analysis showed that increased macrophage infiltration as well as M2 polarization in Oral Leukoplakia is associated with malignant transformation to OSCC (6). Oral Lichen Planus (OLP) showed a different immune infiltrate compared to OL. There was a trend towards increased IL-23R expression and macrophage infiltration as well as a significant increase in CD163 cell count. This indicates an even stronger shift to M2-like macrophages in OLP lesions compared to OL. A previous study showed a significant higher number of CD68 and CD163 positive macrophages in OLP cases compared to Oral Lichenoid Lesions cases without clinical diagnosis of Lichen (22). The Oral Lichen cases analyzed in the current study all showed clinical signs of OLP besides the histologic criteria allowing the clinical diagnosis. A further analysis showed an increase in PD-L1 and the macrophage marker IDO in Oral Lichenoid Lesions and OLP cases compared to controls (23). A further investigation indicates an increase in the expression of the M2 macrophage marker CD163 in dysplastic OLP and Oral Lichenoid Lesions compared to non-dysplastic ones (24). These data indicate a possible role of macrophages in the pathogenesis and malignant transformation of Oral Lichen Planus.

A substantial body of evidence highlights the connection between chronic inflammation and the increased risk of malignant transformation in the affected oral epithelium. Periodontitis has been linked to a higher risk of developing chronic systemic conditions, including autoimmune diseases and various types of cancers (25). Moreover, targeting the IL-23/Th17 axis has been proposed as a potential therapeutic strategy for managing periodontal disease (26). In this regard it is interesting that we could observe an increase of IL-23R expression from healthy Controls to Oral Leukoplakia and to OSCC. OLP samples showed the highest IL-23R expression levels of all analyzed non-malignant samples. IL-23R seems to be associated with the development of cancer. Several genetic polymorphisms of the IL-23R were shown to be associated with and increased or reduced risk of cancer development (8). However, the mechanism by which IL-23 signaling could contribute to cancer development is not clear and could depend on environmental factors and differ in different organs. In esophageal cancer IL-23 signaling seems to contribute to radioresistance. In Helicobacter Pylori associated gastritis an increased IL-23R expression was found. In gastric cancer, higher expression levels of IL-23R were found and its expression was seen to be positively correlated with tumor size and poor clinical prognosis (8). Mechanistically, IL-23R signaling was shown to interfere with the antitumor function of NK cells by blocking their IFN-γ and perforin-mediated effects (8). In addition, it is believed to promote angiogenesis and reduce the infiltration of CD8 + T-cells into tumor tissue (8). As the OLP cases in our study showed a high IL-23R expression and IL-23 inhibitors are clinically available (27), the potential role of anti-IL-23 therapy should be investigated in OLP and OL potentially malignant transforming oral lesions. As we identified a high number (40%) of CD163+IL-23R+ double-positive M2-like macrophages of all IL-23R positive cells with macrophage marker expression, a possible therapeutic inhibition of IL-23 signaling could eventually also have inhibitory effects on M2 macrophages which are believed to contribute to OSCC initiation and progression (6, 7).

The presence of lymph node metastases (N+) is one of the most relevant parameters for aggressiveness and unfavorable prognosis in OSCC (7, 28). Our current data indicate an increase of all analyzed macrophage markers in N+ cases compared to N0. This is consistent with previous data in a collective of early OSCC (7). CD163+ tumor-associated macrophages (TAMs) play a significant role in the progression and microenvironment of oral tumor lesions. CD163 is a specific marker for macrophages that are polarized toward an M2-like phenotype, which is generally associated with anti-inflammatory responses and tissue repair (29). In the context of oral cancer, CD163+ TAMs can contribute to tumor progression through various mechanisms. CD163+ TAMs often exhibit immunosuppressive characteristics, secreting cytokines and factors like IL-10 and TGF-β that help to dampen the immune response. This can prevent effective immune surveillance and allow the tumor to evade detection by the body’s defense mechanisms. CD163+ TAMs contribute to the overall immunosuppressive tumor microenvironment by interacting with other immune cells, such as T-cells and DCs, further suppressing the anti-tumor immune response and promoting a microenvironment favorable for cancer cell survival and progression (29).

Comparing OSCC resection specimens after IT with anti-PD1 with resections without IT there was a significant increase in CD11c and CD163 cell density in cases after IT. Previous data also hint at an increase in T-cell infiltration as well as PD-L1 expression in response to anti-PD1 therapy (12, 30). These results indicate a generally increased density of myeloid cells and T-cells in response to anti-PD1 IT in OSCC samples. As there was also a significant increase in the density of CD163 positive M2-like macrophages in OSCC resection samples after IT, a possible contribution of M2 macrophages to resistance towards neoadjuvant IT could be involved. This is of special importance considering the increasing importance of neoadjuvant anti-PD1 IT in OSCC. Therefore, the influence of anti-PD1 IT on immune cell infiltration should be better investigated and strategies like macrophage modulation or IL-23 inhibition should be considered in combination with anti-PD1 IT.

## Limitations of the study

The current study was intended as a pilot-study to get insights into macrophage composition and IL-23R expression in oral inflammatory and potentially malignant diseases. Therefore, the number in each group is low. Even though significant differences were detected between the analyzed pathologies. Further studies in larger cohorts are needed to verify these results.

Another limitation is the specificity of the available macrophage markers. For example, CD11c is used to identify both M1-like macrophages and dendritic cells, as described above.

## Conclusion

The current pilot study could provide evidence that there are relevant differences in macrophage infiltration and polarization as well as IL-23R expression in different inflammatory and potentially malignant disorders of the oral mucosa as well as in different groups of oral cancer depending on disease staging as well as the application of immunotherapy. As IL-23R expression was upregulated in Oral Leukoplakia and Oral Lichen Planus, the therapeutic potential of already clinically available anti-IL23 therapy should be further investigated in these oral lesions. Methodically, we could show that creation of a single TMA slide with a duplex punch of each tissue of interest and application of an immunofluorescence multi-staining followed by digitalization and AI-assisted cell couniting is suitable for an explorative analysis of different tissue groups.

## Data Availability

The raw data supporting the conclusions of this article will be made available by the authors, without undue reservation.
